# Detecting Clinically Relevant Emotional Distress and Functional Impairment in Children and Adolescents: Protocol for an Automated Speech Analysis Algorithm Development Study

**DOI:** 10.2196/46970

**Published:** 2023-06-23

**Authors:** Yared Alemu, Hua Chen, Chenghao Duan, Desmond Caulley, Rosa I Arriaga, Emre Sezgin

**Affiliations:** 1 TQIntelligence, Inc Tucker, GA United States; 2 Department of Psychiatry and Behavioral Sciences Morehouse School of Medicine Atlanta, GA United States; 3 School of Electrical and Computer Engineering Georgia Institute of Technology Atlanta, GA United States; 4 The Abigail Wexner Research Institute at Nationwide Children’s Hospital Columbus, OH United States

**Keywords:** mental health, predictive modeling, machine learning, artificial intelligence, social determinants of health, speech-recognition, adverse childhood experiences, trauma and emotional distress, voice marker, speech biomarker, pediatrics, at-risk youth

## Abstract

**Background:**

Even before the onset of the COVID-19 pandemic, children and adolescents were experiencing a mental health crisis, partly due to a lack of quality mental health services. The rate of suicide for Black youth has increased by 80%. By 2025, the health care system will be short of 225,000 therapists, further exacerbating the current crisis. Therefore, it is of utmost importance for providers, schools, youth mental health, and pediatric medical providers to integrate innovation in digital mental health to identify problems proactively and rapidly for effective collaboration with other health care providers. Such approaches can help identify robust, reproducible, and generalizable predictors and digital biomarkers of treatment response in psychiatry. Among the multitude of digital innovations to identify a biomarker for psychiatric diseases currently, as part of the macrolevel digital health transformation, speech stands out as an attractive candidate with features such as affordability, noninvasive, and nonintrusive.

**Objective:**

The protocol aims to develop speech-emotion recognition algorithms leveraging artificial intelligence/machine learning, which can establish a link between trauma, stress, and voice types, including disrupting speech-based characteristics, and detect clinically relevant emotional distress and functional impairments in children and adolescents.

**Methods:**

Informed by theoretical foundations (the Theory of Psychological Trauma Biomarkers and Archetypal Voice Categories), we developed our methodology to focus on 5 emotions: anger, happiness, fear, neutral, and sadness. Participants will be recruited from 2 local mental health centers that serve urban youths. Speech samples, along with responses to the Symptom and Functioning Severity Scale, Patient Health Questionnaire 9, and Adverse Childhood Experiences scales, will be collected using an Android mobile app. Our model development pipeline is informed by Gaussian mixture model (GMM), recurrent neural network, and long short-term memory.

**Results:**

We tested our model with a public data set. The GMM with 128 clusters showed an evenly distributed accuracy across all 5 emotions. Using utterance-level features, GMM achieved an accuracy of 79.15% overall, while frame selection increased accuracy to 85.35%. This demonstrates that GMM is a robust model for emotion classification of all 5 emotions and that emotion frame selection enhances accuracy, which is significant for scientific evaluation. Recruitment and data collection for the study were initiated in August 2021 and are currently underway. The study results are likely to be available and published in 2024.

**Conclusions:**

This study contributes to the literature as it addresses the need for speech-focused digital health tools to detect clinically relevant emotional distress and functional impairments in children and adolescents. The preliminary results show that our algorithm has the potential to improve outcomes. The findings will contribute to the broader digital health transformation.

**International Registered Report Identifier (IRRID):**

DERR1-10.2196/46970

## Introduction

### Background

The increase in the rates of mental health problems among the pediatric population predates COVID-19. According to the Substance Abuse and Mental Health Services Administration and US Department of Health and Human Services Centers for Disease Control and Prevention [1]:

Between 2016 and 2020, the number of children ages 3-17 years diagnosed with anxiety grew by 29% and those with depression by 27%. In 2020, suicide was the second leading cause of death for young people aged 10-14 and 25-34, and among the top 9 leading causes of death for people ages 10-64.

There has been an increase in suicide rates among children and adolescents [2], especially in low-income communities [3]. The meta-analysis of 29 studies with a total of more than 80,000 participants indicated an increase in emergency room visits by 22.3% and reported that 1 out of every 4 adolescents is experiencing clinically relevant depressive and anxiety symptoms, which was double the rate of prepandemic levels [4].

The need to focus on young people is partly related to the onset of mental disorders, which usually starts during a person’s early developmental stages, and almost half of all mental health conditions are present before 14 years of age. Trauma plays a role in more than three-quarters of youth experiencing mental health disorders [5]. The early onset of the disease for Black, Indigenous, people of color, and other marginalized communities is related to the high rate of the experience of trauma, which is generational, and partly due to a history of discrimination, poverty, and poor access to quality mental health care. One way to account for the incidents and the severity of trauma is the use of Adverse Childhood Experiences (ACE). ACE scores tend to be higher for families from lower socioeconomic status [6]. ACE identifies a more expensive account of trauma, including the role of divorce and parental incarceration, and substance abuse. ACE score of 4 or more is likely related to symptoms of trauma, including aggression, self-harm behavior, posttraumatic arousal, and inattention, as well as neurobiological, developmental, and social delays [7]. Consequences across the lifespan include disrupted neurodevelopment and socioemotional and cognitive impairment. ACE also exacerbates the chances of future drug abuse, poor physical health, and early chronic disease, disability, and mortality [8].

The current behavioral health care system is falling short in addressing the crisis in the mental health needs of children and adolescents. In addition to the shortage of health care providers, the accurate diagnosis of mental disorders by humans requires extensive training, experience, and resources. Therefore, low-resourced communities have been experiencing a severe lack of experienced mental health providers, while the need for mental health services and the severity of patients remains disproportionately high. There is a need for technology-based and accessible tools for early intervention mechanisms for emotional distress in children and adolescents [9]. As a marker of emotional distress, speech or voice indicators have been used to understand the state of the mood and condition in health care [10]. Previous studies have already underlined the value of speech-based detection mechanisms in mental health. In earlier works, Scherer et al [11] followed 379 patients. They found that acoustic and linguistic measurements from voice samples achieved 85%-93% accuracy in classifying patients as suicidal, mentally ill but not suicidal, or neither. Mota et al [12] used a 2- to 5-minute free speech session and voice analysis method to predict (with 100% accuracy) whether an individual is at risk of first-episode depression. These examples and literature show the potential of speech analysis in detecting psychological distress and mental health issues [13-15]. Speech-based digital health technologies can assist health care providers in identifying children in need and aid in the delivery of appropriate and timely services to families in the community [16]. Specifically, such mechanisms can be used for early intervention, which can delay the onset and reduce the probability of developing the disorder [17]. Such prevention and mental health interventions can divert this population from future mental health issues and other high-risk behaviors [18].

In this study, we aim to develop and test a speech analysis algorithm to detect clinically relevant emotional distress and functional impairments in children and adolescents with diagnosable mental health disorders.

### Theoretical Background

#### Archetypal Voice Categories

A significant problem for identifying speech emotions is the need to classify a collection of appropriate emotions to be identified as automatic emotion recognizers. Linguists have identified inventories of the emotional states that are occurring the most in our lives [19]. Several scholars agree with the “palette principle,” which states that any emotion can be broken down into primary emotions, such as how each color is a mixture of simple colors. In this framework, the primary emotions are anger, happiness, fear, neutral, and sadness. These are the most visible and distinct feelings in our lives, and therefore, these emotions are called archetypal [20]. Given the role of emotions in human communication and decision-making, intelligent human-machine interfaces must respond appropriately to human emotions [20].

Automated emotion recognizers can be seen as devices assigning emotional states to category labels. The detection and identification of emotions in speech involve not only signal processing and interpretation methods but also psychological, linguistic, and emotional interpretation [20]. As a result of the rich voice data that can be identified, the focus on archetypal emotions—anger, happiness, fear, neutral, and sadness—is usually justified as a way of making finer distinctions [21]. On this premise, 2 forms of knowledge are included in the communication structure of human speech. The first type is more direct and communicates using spoken sentences. The second type is more passive and uses implicit, nonverbal signals to convey the message. We focus on understanding negative and nonnegative emotions from implicit and nondirect voice signals. This form of communication represents emotional distress and can help identify human emotions at a deeper level.

#### Theory of Psychological Trauma Biomarkers in Speech

Among all the biomarkers under development, speech stands out as an attractive candidate because it is affordable, noninvasive, and nonintrusive. Speech has the potential for applicability to a cohort of patients with various emotional or behavioral disorders from multiple cultural and linguistic backgrounds [20]. This capability is achieved by selecting only prosodic and acoustic features commonly existing in human speech and using them for predicting everyone's emotional distress and negative emotion [19]. This speech clustering emotion is assisted initially by traditional clinical diagnosis and at least 3 scientifically validated surveys: ACE, Patient Health Questionnaire 9 (PHQ-9), and Symptom and Functioning Severity Scale (SFSS) [22].

The link between trauma or stress and voice types is related to changes in the autonomic nervous system, including the disruption of speech-based characteristics partly due to muscle tensions. For example, evidence points to a connection between neural activity in the gamma-aminobutyric acid neurotransmitter, susceptibility to depression, and changes in muscle tonality [23]. Changes related to muscle tonality produce distinct utterances, thereby indicating the presence of negative mood or depressive symptoms [24]. Additionally, emotional states have a temporal structure. For example, people with bipolar depression or chronic anxiety may be in intense emotional states for months or years or have negative emotions for weeks [25]. On the other hand, emotions such as anger and excitement may be transient and last only for a few minutes. Emotions, therefore, have a broad meaning and a narrow sense. Overall, meaning reflects the long-term emotions underlying it, and the narrow sense applies to the mental stimulation that motivates people to behave in the short term [26]. To correctly identify the level of emotional distress, the algorithm needs to identify the underlying emotions of the behavioral/emotional disorder severity.

## Methods

### Participants

Participants are recruited from 2 mental health care centers that focused primarily on children and adolescents from low-income communities. The participants receive mental health services paid for by Medicaid, the Children’s Health Institute Program, and the Department of Children and Family Services. The participants are children aged 5-18 years and those who have been referred for behavioral health services through schools, DJJ, and child protective agencies.

### Ethical Considerations

The study received ethical board approval for a multisite pilot involving children and adolescents. We will receive written and informed consent from participants. The activities for patients enrolled in the study include data collected using multiple surveys and a voice sample. The surveys include structured and unstructured voice samples based on a reading selected by the research team. All the study contents are approved by the ethical board.

### Data Collection

Using a software application, we use the TQI app [27] to collect voice samples from youth receiving mental health and family preservation services at the point of care. The app and data collection process include the concurrent administration of a scientifically validated, SFSS [28]. The patient's emotional disorder severity is confirmed based on the scores of the SFSS, ACE, and PHQ-9, as well as the patient’s diagnosis and other clinical information from the patient's treatment history. For every voice sample collected, the pilot has a diagnosis or multiple, current procedural terminology code, and the therapist’s clinical input. These data serve to categorize voice sample data into distinct categories and train voice-based machine learning (ML) algorithms to distinguish children in need and at high risk. The innovation is focused on families with low socioeconomic status, in whom ACE are common.

Data are collected by trained therapists (n=15). An Android mobile app is used to collect speech samples, SFSS, PHQ-9, and ACE scale responses. Each therapist receives a 5-hour in-person training about app installation and access, data (SFSS, voice sample) collection using the app, compliance and parameters, the process of the reward program, communication, and real-time technical support. Data are collected from patients every week as part of the mental health or other family-based intervention visits. Visits occur in a suitable (private) location at home (including foster and group homes) or school as part of a state’s commitment to access services. Data are collected virtually as part of the shift in services secondary to the COVID-19 pandemic. Initially, identified target patient group demographics are shared in Table 1.

Voice samples are collected using the TQI app. To elicit speech for recording and analysis, patients are asked to choose from a subset of readings. Patients’ reading is recorded for up to 90 seconds—a clock feature in the app signals when the time is up. Voice samples are stored in a separate deidentified database that will allow 3 to 4 psychologists to label the voice sample independently. SFSS, PHQ-9, and ACE responses are collected using the same app. The app scores the surveys immediately and makes the results available to the therapist to share with their patients if it is clinically appropriate; the availability of the data in real time is intended to close the gap in transparency, accountability, and family engagement.

**Table 1:** Identified target patient group demographics.

Characteristics		Values (N=420), n (%)
**Gender**		
	Female	136 (32)
	Male	147 (35)
	No gender disclosed	137 (33)
**Ethnicity**		
	Hispanic	115 (27)
	Caucasian	66 (16)
	Black	93 (22)
	Asian	2 (0.5)
	No ethnicity disclosed	144 (34)

### Data Processing

#### Data Preparation

Preprocessing speech samples, that is, segregating the voiced region from the silent and unvoiced portion of an utterance, is a crucial step in developing reliable voice analysis. Most of the speech-specific attributes are present in the voiced part of speech, and our algorithm learns emotion only based on the voiced portion as opposed to the whole segment. To extract the active voice from our samples, an open-source voice activity detection (VAD) algorithm called “WebRTC VAD” [29] is used in preprocessing. WebRTC VAD is a power-based VAD algorithm that extracts the features such as spectral energy and zero crossing rate during a short time frame of 30 milliseconds and feeds them into a decision tree trained by a large voice data set to decide the voice activity. As the clinical data collected from the app contains different levels of noise, we chose a high aggressiveness for our VAD in order to clean the data into voiced utterances.

#### Feature Extraction

Feature analysis is made by applying a shifting Hamming window of 25.6-minute length frames of the speech signal. Within each frame, the signal is approximately stationary. We then estimate the spectrum by calculating the discrete Fourier transform for each segment with N-point FFT (Fast Fourier transform) of 256 samples. For each analysis frame, we extract time domain feature, spectral features, and Mel-frequency cepstrum coefficient.

The time domain features extracted are zero crossing rate, energy, and entropy. Pitch and energy information are the most prominent features, as they can identify emotion based on the tension of vocal folds and subglottal air pressure. The time elapsed between 2 successive vocal fold openings is called a pitch period, while the vibration rate of the vocal folds is the fundamental frequency. Smaller pitch periods, that is, higher velocities of airflow, are associated with emotions like happiness, while longer pitch periods are linked to harsher emotions like anger. The spectral features extracted are entropy, spread, flux, and roll-off. The alterations in estimations of spectral spread over the frequency range and entropy in speech have been proven to be strongly linked to the diagnoses of clinical depression [30].

The Mel-frequency cepstrum coefficient extracted from the speech is a representation of linear cosine transform of a short-term log power spectrum of the speech signal on a nonlinear Mel scale of frequency. The incorporation of these coefficients in the feature vector is useful in the identification of paralinguistic content in speech. This extraction is of the type where all the characteristics of the speech signal are concentrated on the first few coefficients. A probability model algorithm is used to classify speech and to derive a prediction of the speaker’s emotions.

#### Emotional Frame Selection

Feature extraction procedures produce a quantified collection of attributes. However, emotion recognition is still challenging as we do not understand the correlation between certain features and certain emotions. To better analyze and understand the correlation, we use a Gaussian mixture model (GMM) to perform frame selections. The key intuition is that emotions are usually more concentrated on certain words. While we are not studying emotion from a semantics perspective, it does help us to locate emotionally rich frames and study their features.

We perform GMM clustering on frame features in an utterance. The cluster means are visualized with multidimensional scaling, projecting the Euclidean distance between all clusters onto 2D map. We repeat the process for all emotion categories except neutral. Drawing inspiration from intuition, we deem frames close to the origin on the projection map to be less discriminative and frames on the outer ring to be more discriminative. By using the selected frames, inputs become more condensed hence boosting the classification accuracy.

### Machine Learning Models

#### Gaussian Mixture Model

Our first model takes an unsupervised parametric clustering approach. The GMM is a parametric probability density function consisting of several single Gaussian distributions [31]. It is an unsupervised soft clustering technique that is widely used for speech and speaker classification, audio classification, audio segmentation, and so forth. A GMM approach was used, where speech emotions were modeled as a mixture of Gaussian densities. The model is parameterized by 2 types of values: the mixture component weights and the component mean and variances or covariances. For a model with K components, the kth component has a mean vector of length k and k × k dimensional covariance matrix. The mixture component weights are defined such that the total probability distribution normalizes to 1. If the component weights are not learned, they are viewed as an a priori distribution over components. If they are learned, they are the a posteriori estimates of the component probabilities given the data.

In the first step (expectation or E), the expectation of the component assignments for each data point is calculated. In the second step (maximization or M), the expectations calculated in the E step are maximized. This iterative process repeats until the algorithm converges, giving a maximum likelihood estimate. Alternating between which values are assumed fixed or known, maximum likelihood estimates of the nonfixed values can be calculated efficiently.

To ensure GMM’s performance, we extract features from audio samples that have a satisfying discriminate capacity. We apply Fisher discriminant ratio [32] for the feature selection process. Fisher discriminant ratio is a significant measure for features to be used in classification. For our task, Fisher discriminant ratio indicates how separable the clusters for different emotions are. The top features include energy below 250 Hz, median and minimum of F1 formant frequency, a median of F0 formant frequency, and the variation of the duration of energy plateaus (represents speech rate). Other features used in our analysis include linear predictive coding coefficients and Mel-frequency cepstrum coefficients. Linear predictive coding coefficients is a robust, reliable, and accurate linear representation of speech that takes into consideration the vocal tract and glottal pulse characteristics of voiced speech [33]. Mel-frequency cepstrum coefficients is extracted on a nonlinear scale of frequencies that are altered to closely resemble human hearing capabilities [34]. As we are aiming at children's stress detection with varying age differences across all children patients, it is important to note the difference in the vocal cord between gender and age groups.

There are 2 general approaches for GMM parameter estimation methods: the maximum likelihood approach and expectation-maximization (EM) estimation [35]. The ML method constructs a most likely model to fit for all of the current observations. Since there is no way to ensure unbiased data collection regarding mental health, the ML method poses an unrealistic assumption on the data set. Comparatively, EM is a far superior method to cope with this so-called missing data problem, where we assume only a subset of the features is observed. The EM algorithm consists of 2 iterative steps. Expectation or the E-step made an initial guess for the model parameter concerning missing data, using current estimates, and conditioned upon observed data. The maximization (M-step) then provides a new estimate increasing the above expectation.

The entire iterative process repeats until the algorithm converges, giving a maximum likelihood estimate. Intuitively, the algorithm works because knowing the component assignment Ck for each xi makes solving for model parameters easy while knowing the model parameters and makes inferring the probability of point xi belonging to component Ck easy. The expectation step corresponds to the latter case, while the maximization step corresponds to the former. Thus, alternating between which values are assumed fixed or known, maximum likelihood estimates of the nonfixed values can be calculated efficiently.

#### Recurrent Neural Network and Long Short-Term Memory

Our second model takes on a different approach to learning, where it employs a supervised learning scheme. The primary motivation for using a recurrent neural network is the idea that they can connect previous information to the present, such as using features extracted from previous frames of the signal to understand the present frame [30]. A long short-term memory network is a special kind of recurrent neural network (RNN) capable of learning long-term dependencies. It uses memory cells and gates to control whether the information is memorized, output, or forgotten [30]. The gating mechanism is used to control information flow by pointwise multiplication. There is a cell to memorize information within the unit. The long short-term memory (LSTM) incorporates cell states at several different time points prior to the current frame using a linear combination, which is computed using an attention mechanism where the weights of data points are learned. This configuration implies an assumption that the current state depends on the state of the previous time step. Thus, emotion patterns are learned by not only correlating the features to the labels independently but also taking the change in features in time into consideration.

Recurrent neural network is a potent tool to extract features in speech recognition, audio scene detection, and so forth. Unlike artificial neural networks, RNN forms a directional graph along a temporal sequence, which registers temporal information throughout the learning process. RNN has proven to be extremely powerful in speech- or audio-related tasks [20,21,23,24] due to the temporal nature of speech. Emotion poses a similar temporal feature to speech, so we wish to create an RNN-based neural network for our task. There has been some research using RNN/LSTM networks for emotion classification. LSTM units are capable of learning long-term dependencies and avoid vanishing gradient problems. With more and more clinical data flooding our database, we are quite positive about what RNN can achieve for our task.

## Results

### Overview

The institutional review board approved the study in July 2021. Recruitment and data collection for the study were initiated in August 2021 and are currently underway. The study results are likely to be available and published in 2024.

### Testing With a Public Data Set

The proposed models are first trained and tested on large public voice data sets to validate the model’s ability of emotion classification. These data sets are the Toronto emotion speech set, Ryerson audio-visual database of emotional speech and song, and Surrey audio-visual expressed emotion, which have simulated emotions provided by professional actors [36]. The voice samples are split into training and testing sets, with a ratio of 85:15 for each of the 5 emotions. The models are trained both with utterance level features and with emotion frame selection to demonstrate the effectiveness.

Based on the results (Table 2), GMM with 128 clusters shows an evenly distributed accuracy across 5 emotions. With utterance level features, GMM achieves an accuracy of 79.15% in total and with frame selection of 85.35%. This demonstrates that GMM is a robust model for emotion classification of all 5 emotions, and emotion frame selection enhances the accuracy of all emotions, which is significant for scientific evaluation. The RNN-LSTM model also shows an evenly distributed accuracy over the emotions, with 79.6% overall accuracy on utterance level and 86% overall accuracy on emotional frames. With emotion frame selection, accuracy on emotions is enhanced but for fear.

**Table 2:** Accuracy testing results (%) for emotions with the public data set.

Emotions	GMM^a^ utterance (%)	GMM frame selection (%)	RNN^b^ utterance (%)	RNN frame selection (%)
Neutral	81.68	89.97	86	87
Happy	75.77	82.50	76	88
Sad	73.61	80.10	73	88
Angry	82.52	87.27	84	96
Fear	81.6	85.89	79	71
Overall	79.15	85.35	80	86

^a^GMM: Gaussian mixture model.

^b^RNN: recurrent neural network.

### Clinical Data

During this study, we plan to collect and test our model with a clinical data set. Due to the potential variance of the sample size of available clinical data and the richness of emotions that may differ in the pediatric population, training with clinical data is necessary with GMM. For the public data set, our models yield unbiased classification accuracy across all emotion categories. GMM and RNN results are on par, and both could take advantage of the frame selection process to boost accuracy by 6.2% and 6.4%, respectively. Yet, GMM and RNN-LSTM approaches traditionally require a large amount of training data to achieve the best results. Therefore, the clinical data set is aimed to be diverse and large enough to meet the requirement for the training data set. Nevertheless, the results show that our approach is effective for adult human emotion recognition.

For the clinic data set, the performance drop in children’s emotion recognition is expected, especially for the data trained on adult speech and tested on children's speech. It may demonstrate the difference between adults’ and children’s vocal quality. For models trained on clinical data sets, the performance depends on data sample size and quality. In addition, children's voice quality between 6 and 18 years of age is different across gender as well as age groups. It indicates that the training data set is required to be clustered into separate groups. Another parameter to consider is the natural bias in the data set. Since voice samples are collected from patients, they could be biased toward negative emotions, especially fear, anger, and sad categories.

## Discussion

### Principal Findings

Our study aims to develop and test a speech analysis algorithm to detect clinically relevant emotional distress and functional impairments in children and adolescents with diagnosable mental health disorders. More specifically, the study proposal focused on developing an ML model for a feature extraction that is used for discriminating clinically relevant emotions from speech utterances. The ML model we built also achieved its goal of emotion classification and frame selection for pediatric patients with mental health disorders.

The clinical voice samples provided a wealth of information that can indicate mental status and overall emotional functioning [10], and our study is building on top of earlier evidence on predicting mental conditions [11], including depression and emotional distress [13-15]. Therefore, we anticipate that our study findings will contribute to the literature in terms of understanding the current landscape and building evidence to develop an automated speech analysis algorithm to detect emotional distress and functional impairment.

Even though the study data collection is ongoing, preliminary testing with a public data set showed promising results. The study developed a robust emotion recognition algorithm for 5 classes of emotion based on open-source data sets with 79.14% accuracy using probabilistic models. Compared to the literature, we have promising scores in terms of accuracy with the public data set [11]. A GMM-based speech emotion classification algorithm is used with 3 public adult speech corpora.

Developing a more accurate model requires addressing the problem of biased samples; currently, all the voice samples are collected from youth in mental health services with known mental health diagnoses. The average score of ACE for this sample population (N=420) is 5 out of 10; less than 13% of the US population has an ACE score of 4 or higher [37]. Furthermore, some of the youths display regional Southern American accents, African American Vernacular English, as well as accents related to the fact that English is not their first language. While this gives us the opportunity to build a unique clinical data set from youth from low-income communities, a skewed data set may decrease the accuracy of the algorithm to detect and score the severity of negative emotions.

### Limitations

The study presents several limitations. First, the quality and variability of speech data, including background noise and speech disorders, can affect the accuracy and reliability of biomarkers. Second, the sample population may not be representative to the greater US population, limiting the generalizability of findings. Finally, legal and regulatory considerations, including privacy rules and compliance with regulations, must be reviewed based on the state and jurisdictions, and may limit the use of the algorithm. The latter must be considered to protect participants and ensure the lawful use of data.

### Implications and Future Work

In the United States, there are several bottlenecks in the current system of mental health care that technology can address immediately. First is a quick and accurate measurement of the severity of the mental health problem, keeping in mind that all planned interventions depend on the accuracy of symptom severity. Technology, like voice and artificial intelligence, that can make objective measurements of severity can be integrated with limited disruption to the workflow. They can play a significant role in triaging youth to the appropriate level of services proactively and effectively. As we transition to a knowledge-based economy and adjust to the digital industrial revolution, the mental health system of care can no longer afford to resist technology as one part of improving treatment outcomes While automation is routinely adopted in other sectors of the economy, the semimanual workflow of the current mental health system of care accounts for inefficiency in care and provider burnout. For example, mental health providers spend 40% of their time documenting, equivalent to 2 days out of the 5-day workweek that they cannot provide patient services.

Artificial intelligence, automation, and data analytics will be an integral part of the future of mental health services. Adoption of this technology will require the design of user-friendly interfaces, such as the TQI app, that can help mitigate clinician technophobia [38]. Because of the anticipated lack of qualified providers, health care organizations and school systems need to adopt innovation to address quality and cost. As a result, we expect to see the prevalence of innovative technologies continue to rise as payers and other stakeholders demand evidence-based services and data demonstrating treatment outcomes. The shift to value-based care is from payment based on service provided to payment based on outcomes. The providers that will thrive in this new service landscape must use technologies to improve clinical accuracy, workflow productivity, and efficiency. This will help service organizations not only to identify and track high-risk children but also to improve treatment outcomes. A significant percentage of patients in child protective services and covered by Medicaid and other state expenditures come from low-resourced communities. Such solutions will mitigate the severe shortage of highly trained mental health providers in these communities and address health outcome disparities.

The current student-to–school counselor ratio for most school systems is 300 students to 1 counselor. Some schools have a contractual agreement with virtual behavioral health providers to lessen this burden for their staff. The technology could play an influential role in integrating services and breaking down silos. Collaboration between mental health care providers, pediatric primary care, schools, and other stakeholders has been shown to be effective in the early identification and treatment of mental health conditions.

### Conclusions

The integration of digital mental health innovations is crucial in addressing the ongoing mental health crisis among children and adolescents. Speech-based digital biomarkers that can be collected via a user-friendly interface hold promise for identifying emotional distress and functional impairments in this population. The findings will contribute to the broader digital health transformation and pave the way for proactive and collaborative mental health care practices.

## Appendix

Multimedia Appendix 1 Peer review reports.

## Abbreviations

ACE: adverse childhood experiences
EM: expectation-maximization
GMM: Gaussian mixture model
LSTM: long short-term memory
ML: machine learning
PHQ-9: Patient Health Questionnaire 9
RNN: recurrent neural network
SFSS: Symptom and Functioning Severity Scale
VAD: voice activity detection

## Data Availability

Data sharing is not applicable to this study as no data sets were generated or analyzed during this study. Preliminary work was completed using a public data set, and the data set is cited in the manuscript.
